# Characterization of Metallic Off-Flavors in Drinking Water: Health, Consumption, and Sensory Perception

**DOI:** 10.3390/ijerph192416829

**Published:** 2022-12-15

**Authors:** Susan Mirlohi

**Affiliations:** Department of Public Health, California State University, Fresno, CA 93740-8031, USA; susanmirlohi@csufresno.edu; Tel.: +1-559-278-7024

**Keywords:** metallic flavor, iron, copper, lipid oxidation, artificial saliva, water consumption, sensory perception

## Abstract

Characterization of taste- and flavor-producing metals, namely iron and copper, in drinking water is a multifaceted subject. Both metals are essential nutrients, can be toxic, and are known to produce unpleasant tastes and flavor sensations in drinking water. Ingestion of trace metal contaminants through drinking water is a probable source of human exposure. Biochemical mechanisms of metallic flavor perception have been previously described; however, less is known about how variations in salivary constituents might impact individuals’ sensitivities to metallic flavors and beverage consumption behaviors. This research presents findings from in vitro experiments, using artificial human saliva, to better understand the role of salivary lipids and proteins on metallic flavor production as measured by biomarkers of metal-induced oxidative stress. The results indicate that metal-induced lipid oxidation, as measured by thiobarbituric acid reactive substances (TBARS), is dominated by salivary proteins, is slightly inhibited in the presence of salivary nitrite, and is detectable by the TBARS method at and above respective concentrations of 9 µM (0.5 mg/L) and 90 µM (5 mg/L), which are both above the aesthetic standards for iron (0.3 mg/L) and copper (1.0 mg/L) in drinking water. Preliminary study with human subjects indicated that reduction in metallic flavor sensitivity, as measured by the best estimate flavor threshold for ferrous iron among 33 healthy adults aged 19–84 years old (22 females), corresponded with reduced drinking water consumption and increased caloric beverage intake among older subjects (>60 years), as determined by a validated self-reported beverage intake questionnaire. These findings provide insights for further research to examine how salivary constituents can impact humans’ sensory abilities in detecting metallic off-flavors in water, and how reduced metallic flavor sensitivity may influence beverage choices and drinking water consumption.

## 1. Introduction

Transition elements, iron and copper, are recognized as two major flavor-producing metals in drinking water [1,2]. They are associated with many taste complaints among drinking water consumers as indicated in a survey of North American utilities [3]. They can be caused by corrosion of iron or copper [4,5] pipes in water distribution systems or occurrence of ferrous iron in groundwater [6,7]. In human sensory studies, perceived flavors of iron and copper have been characterized as metallic, salty, bitter, and astringent [8,9,10,11]. Ferrous iron typically produces the strongest metallic flavor, followed by cupric and cuprous salts, while bitterness and astringency are typically associated with the taste of copper [12,13]. In its metallic nanoparticle form, oral exposure to iron is believed to produce a weaker metallic flavor than that of ferrous iron based on indirect measure of its metallic flavor intensity as measured by lipid oxidation using in vitro experiments with actual human saliva [14].

Interestingly, metal-induced lipid oxidation in the oral cavity, as measured by the method of thiobarbituric reactive substances (TBARS) in human saliva after oral exposure to iron and copper, has been linked to the mechanism by which humans are able to detect the metallic flavor of iron and to some extent copper due to the release of volatile and odorous by-products of lipid oxidation in the oral cavity [8,10,12]. The metallic flavor is thought to be perceived through retronasal detection of aromas associated with odorous aldehydes, such as hexanal, heptanal, and 1-octen-3-one produced due to iron-induced LO reactions [15,16,17], since nose closure results in diminished or loss of this metallic flavor perception [10]. Within the oral cavity, lipid oxidation is caused by free radicals attacking lipid membranes [12] and salivary lipids that are produced through the salivary glands [18]. Metals act as catalysts in the free radical processes that break down polyunsaturated fats [19], while salivary nitrite has been shown to inhibit lipid oxidation in meat products under acidic conditions [20]. Salivary proteins also play an important role in the interaction of saliva with metals, and thus may influence flavor perception [9]. For example, interactions of the salivary protein alpha-amylase with copper impacts its solubility, and thus contributes to the sensation of astringency associated with the flavor of copper in drinking water [21]. Another major salivary protein, mucin, has also been shown to have a high affinity for copper [22]. In biological fluids, binding of lipid oxidation by-products, such as malondialdehyde (MDA) and 4-hydroxynonenal (4-HNE), to proteins has been identified as an indirect cause of protein oxidation, which also contributes to oxidative stress [23,24]. In food systems, some proteins demonstrate antioxidant activities through their metal-binding properties, which indirectly inhibit iron-catalyzed lipid oxidation reactions [25]. Salivary fatty acids are also known to contribute to fat flavor perception through differences in salivary lipolytic activity among individuals with varying sensory thresholds for fatty acids [26]. Additionally, total salivary antioxidant capacity, as a measure of salivary oxidative stress, has been linked to disease, nutritional status, and flavor perception [27]. Salivary enzyme activities, such as lipase and alpha amylase, have been linked to individuals’ diets and food intake, with higher enzyme activity levels in overweight individuals when compared to normal weight individuals; this demonstrated a positive correlational relationship between salivary lipase activity and total fat, protein, and carbohydrate intakes [28]. Salivary protein profiles of individuals have been shown to differ according to hyper- or hypo-sensitivity levels to the bitter taste of caffeine [29]. Variations in individuals’ salivary zinc levels are also believed to play a role in decreased sensitivity to fat flavor perception and increased fatty food consumption [28]. These findings indicate that salivary fluid is an important factor in taste and flavor perception, and its composition can influence food and beverage choices and be indirectly influenced by diet.

Humans vary widely in their sensory abilities and inabilities to detect metallic off-flavors in drinking water, and the potential for toxic level exposure or cause for lack of consumption exists. In the United States, the national drinking water standards established by the Environmental Protection Agency (USEPA) are intended to ensure safety and quality of drinking water for public consumption, as are the standards established by the World Health Organizations [1,2]. Some standards are health-based and regulated by the primary maximum contaminant levels (PMCLs), which are not to be exceeded in potable waters; others are aesthetic-based, such as secondary maximum contaminant levels (SMCLs), recommended to ensure acceptable appearance and palatability of drinking water [1]. Public perception of drinking water safety, while difficult to measure, is known to influence consumers’ behavior. For example, attributes such as taste, odor, and color are often used as indicators of water purity and safety by consumers [30,31,32,33,34]. Among unpleasant flavor attributes, metallic taste is a common consumer complaint [3]. Incidentally, metal contamination of drinking water by copper, which produces a distinct metallic flavor in water, often described as bitter and/or astringent [8,10,11,35], was identified as the source of 27 illness outbreaks in the US since 1971 [36]. Iron, another commonly occurring metal responsible for imparting metallic off-flavor to drinking water [2], is also a common consumer complaint among well and tap water users [3,31,37]. More recently, it has been suggested that the current USEPA secondary standards for some drinking water contaminants, including copper and iron, be revaluated since they may exceed levels necessary for consumers’ acceptance of palatability [38].

Intake of adequate fluid and/or water is vital for the maintenance of human bodily functions, such as cellular metabolism, regulation of body heat, and maintaining the osmolarity of various bodily fluids [39,40,41]. Dehydration has been associated with increased risk of developing kidney stones, urinary tract infections, and bladder cancer, while mild day-to-day dehydration has been associated with fatigue and impaired cognitive performance [40,41,42]. The elderly and infants are typically at greater risk of dehydration [39,43]. Among the elderly, diminished sensation of thirst as well as behavioral reasons such as fear of incontinence and/or disabling conditions associated with aging can contribute to dehydration risk. Age-associated decline in taste functions as well as swallowing disorders have also been recognized as contributing factors to dehydration risk among the elderly [44,45]. According to the United States’ National Health and Nutrition Examination Survey (NHANES), in the period of 2009–2012, American adults, grouped by male and female genders, consumed an average daily amount of 3.46 L (males) and 2.75 L (females) of total water, with plain water and other dietary water (from food and other beverages) representing 30% (males) and 48% (females) of the total beverage intake [46]. Additionally, men and women over the age of 60 consumed less water (2.92 L for men and 2.51 L for women) than younger adults [46]. The United States Institute of Medicine’s Food and Nutrition Board recommends an adequate intake (AI) of daily total water in healthy adult males and females to be 3.7 and 2.7 L, respectively. In spite of this recommendation, the amount of water consumed in a day varies considerably among different age groups and is also varied according to demographics [43].

Since water consumption is vital to bodily functions and plain drinking water intake is encouraged as a healthy beverage in place of calorie containing beverages, it is plausible to consider factors that influence human consumption of drinking water. These factors may include consumer preference for a certain taste or flavor, such as tap, bottled, filtered, or mineral water, as well as the availability of a safe and clean source of water. Consumers’ choice of drinking water may also be driven by their perception of risks associated with a given water source [47,48]. For example, in public water systems, when water utilities experience problems with a certain contaminant of concern, consumers may resort to drinking alternative sources, such as bottled and/or filtered water, due to safety concerns. In fact, this was shown to be the case in studies on Canadian consumers [49]. Variations on individuals’ sensitivities to astringent or bitter flavor-producing foods and beverages, namely vegetables, fruits, and tannin rich beverages such as coffee and tea, have been known to influence consumption [50]. Sensitivity to sweet taste has also been negatively related to consumption, although measures of consumers’ taste preferences have been regarded as better predictors of sweet food and beverage consumption [51]. Additionally, quality of water, including the level of hardness, influenced by mineral content and ferrous iron salt has been shown to influence perception of sweet taste depending on the type of sweeteners [52]. The important role of humans’ basic taste (i.e., bitter, sweet, sour, and umami) functions and sensitivities, which are impacted by genetic, physiological, environmental exposures, and disease outcomes, are widely recognized in relation to influence on dietary intake [53]; however, less is known about the more complex nature of metallic flavor sensation [13] and how human variations in metallic flavor sensitivity might influence water and beverage consumption.

The goals of this study were to compare the levels of iron- and copper-induced salivary lipid oxidation (SLO) in artificial human saliva as indirect measures of their metallic flavor intensities, and to assess how the presence of proteins, fatty acids, and nitrite in saliva may influence metallic flavor production as measured by the TBARS method. Another goal of this study was to examine the beverage consumption pattern in a subset group of healthy adults who were participants in a separate sensory study that assessed their sensitivities to metallic flavor of iron in drinking water [54]. The findings provide additional insights in understanding the role of salivary fluid on metallic flavor production of iron and copper as associated with oral exposure through drinking water. In addition, possible relationships between individuals’ metallic flavor sensitivity and beverage intake levels are identified for future research considerations.

## 2. Methods and Materials

### 2.1. Artificial Saliva Mixture

Artificial saliva was prepared according to the recipes utilized by [26,38]. This contained a mixture of inorganic components, consisting of NaCl (0.1256 g), KCl (0.9639 g), KSCN (0.189 g), KH2PO4 (0.655 g), Na_2_SO_4_ (0.337 g), NH_4_Cl (0.178 g), CaCl_2_ (0.172 g) and NaHCO_3_ (0.631 g), dissolved in 1000 mL of Nanopure^®^ water to make an artificial saliva stock solution. Both 0.216 g of mucin (Sigma-Aldrich, St. Louis, MO, USA; CAS No. 84082-64-4) and 20,000 units, 0.541 g, of α-amylase (Sigma-Aldrich, St. Louis, MO, USA; CAS No. 9001-19-8) were mixed in 100 mL of the inorganic saliva mixture to make the protein-spiked saliva solution. To make the lipid-amended saliva solution, 30 mg of linoleic acid (ACROS, Princeton, NJ, USA, CAS No. 60-33-3) was added to 100 mL of the inorganic saliva mixture. Linoleic acid was used, as it is one of the major fatty acids in oral membrane lipids as well as a major constituent of total salivary lipids [18]. Nitrite-amended saliva solutions contained 250 mM of NO_2_^−^ using sodium nitrite salt (Sigma-Aldrich, St. Louis, MO, USA; CAS No. 237213). Reported concentrations of nitrite in human saliva range from 60 to 1600 mM [55], while total lipid and protein concentrations range from 2.4 to 80 mg/L and 0.6 to 4.0 g/L, respectively [18,56,57,58].

### 2.2. Metal Salts Stock Solutions

The 1.0 g/L iron and copper stock solutions were prepared using ACS grade iron (II) sulfate heptahydrate (Sigma-Aldrich, PA, USA, CAS # 13463-43-g) and copper (II) sulfate pentahydrate (Sigma-Aldrich, PA, USA, CAS # 13463-43-g) in Nanopure^®^ water. Metal solutions were prepared immediately prior to use in testing to minimize air oxidation. The stock solutions were added in microliter amounts to achieve the targeted dosage of iron and/or copper in each of the artificial saliva test solutions described in the next section.

### 2.3. Salivary Lipid Oxidation Experiments with Artificial Saliva

Salivary lipid oxidation (SLO) experiments were conducted on artificial saliva samples that included or excluded different salivary constituents in order to examine the extent of SLO under each condition. Test samples consisted of: (1) artificial saliva (AS) solution that contained only the inorganic constituents noted above, (2) AS solution amended with linoleic acid, (AS + LA), (3) AS solution supplemented with alpha-amylase and mucin (AS + Protein), (4) AS solution supplemented with both proteins and lipid (AS + LA + Protein), and (5) AS solution spiked with proteins, lipid, and nitrite (AS + LA + Protein + Nitrite). Test samples (1 through 5) were separately spiked with ferrous iron and cupric copper at a concentration of 180 mM. Additionally, the test sample AS + LA was treated with varying concentrations of ferrous and cupric salts in order to examine the effect of metal concentration on the level of SLO induced by each metal. The concentration series for both ferrous and cupric consisted of 0, 4.5, 9, 18, 45, 90, 180, and 360 mM. The pH level was measured in each test sample using an ion selective pH electrode (Fisher Accumet). Upon addition of the metals, all test samples were placed in a 37 °C water bath for 15 min to simulate the temperature in the oral environment. The 15-min time was based on a typical salivary flow rate of 5 mL per 15 minutes [59]. At the end of the incubation period, samples were immediately cooled and analyzed for lipid oxidation using the method of TBARS [60]. The TBARS method [60] was modified to work with liquid samples and to enhance readings at low concentrations [61]. Using the modified TBARS procedure, 1 mL of saliva samples and known concentrations of 1,1,3,3-tetramethoxypropane (MDA) standards were each mixed with 2 mL of prepared TBA solution consisting of 0.375% TBA, 0.506% sodium dodecyl sulfate (SDS), and 9.370% glacial acetic acid and digested for 60 min at 95 °C in a water bath. After digestions, samples were immediately cooled in an ice bath, mixed with 2 mL of n-butanol/pyridine mixture (at a 15:1 ratio), and centrifuged for 15 min at a speed of 3000× *g*. The absorbance of the supernatant from each sample and standard was measured with a spectrophotometer at 532 nm. The concentration of TBARS was obtained from the standard curve and absorbance values. The standard curve was developed by running the TBARS method on known MDA standards at 0.03 to 10 μM concentrations. TBARS analysis was run in replicates of four for each sample. Table 1 provides a summary of the artificial saliva test sample identifications and treatment conditions, along with designated abbreviations to be referred to in subsequent sections.

### 2.4. Study Participants

The study was approved by the Institutional Review Board (IRB) at Virginia Tech (IRB Project No. 06-395). Study participants were recruited from the Blacksburg (VA) community, as well as students, faculty, and staff of Virginia Tech by means of paper and email flyers. Subjects were required to have no chronic oral or general health problems, be non-smokers, and not be pregnant. All subjects read and signed an informed consent form in accordance with the approved IRB protocols. A total of 33 (22 females) multinational subjects, ages 19–84 years, participated in the study. Each subject completed a brief questionnaire that provided information on his or her age, gender, smoking, drinking water preference, and general health status. Additionally, each subject completed a more detailed beverage intake questionnaire described in Section 2.5 below. The questionnaire was used to assess the subjects’ daily beverage intake during the previous month. The study questionnaires were completed in-person during the first session of the study, when participants were familiarized with metallic flavor sensation, before sensory threshold testing began.

### 2.5. Beverage Intake Questionnaire

To estimate the mean daily intake of water, sugar-sweetened beverages, and total beverages (in volume), a previously validated beverage questionnaire (BEVQ) was used [62]. The questionnaire consisted of 19 beverage categories and 1 open-ended section for “other” beverages [62]. Scoring instructions for the BEVQ were provided by Hedrick et al. (2010). Briefly, to score the BEVQ, frequency (“how often”) was converted to the unit of times per day and then multiplied by the amount consumed (“how much each time”) to provide average daily beverage consumption in fluid ounces, then converted to units of milliliters (mL). To quantify total sugar-sweetened beverage (SSB) consumption, beverage categories containing added sugars were summed (sweetened juice beverages/drinks, regular soft drinks, sweet tea, sweetened coffee, energy drinks, mixed alcoholic drinks, and meal replacement beverages). For data presentation, beverages not listed under the category of SSB are referred to as “non-SSB” beverages. These included 100% fruit juices, milk, unsweetened or artificially sweetened tea and/or coffee, and alcoholic beverages.

### 2.6. Metallic Flavor Sensory Threshold Determination

The sensory protocol used for individual metallic flavor threshold determinations has been described in a previous publication [54]. Because ferrous iron produces the strongest metallic flavor in drinking water, a threshold study was conducted using ferrous iron. Briefly, for threshold determinations, an ascending concentration one-of-five forced choice test was used [63,64]. Samples were served at 22 to 24 °C in taste-and odor-free 3-oz Solo white plastic cups (Solo Cup Company, Lake Forest, IL, USA) filled with 20 mL of sample and/or control water. Only one sensory session that tested a single ferrous concentration was conducted per day. A single test and concentration per day was necessary to avoid any effect of aftertaste, which is typical of metallic flavor. Subjects were instructed to avoid consuming food or beverages for at least one hour prior to each sensory testing session. Tests were conducted in a quiet room with no distracting odors or sounds. To familiarize subjects with metallic flavor, they were given deionized water with a 20 mg/L iron concentration at their first session, before threshold testing began. At each session, subjects received 5 cups each labeled with a different 3-digit number. Four cups contained deionized water and one contained the ferrous solution. The cups were presented in a random order such that the ferrous solution could be in any of the five positions. Subjects were instructed to taste the samples from left to right without going back, wait 1 min in between samples, and to select the metallic tasting sample and mark it on their score sheet. For a given subject, testing was complete when the subject correctly identified three sequential ferrous concentrations or reached the last and highest concentration approved by the IRB.

“The individuals’ best estimates of thresholds (BET) for metallic flavor of iron were calculated by the geometric mean method. The geometric mean is based on the highest concentration of ferrous iron solution that a participant is unable to taste along with the lowest concentration the participant is able to taste, followed by two other correct choices. The geometric mean is then calculated using the last incorrect ferrous iron concentration and the first correct ferrous iron concentration. For example, for a participant who correctly identifies metallic flavor at 0.05, 0.1, and 0.25 mg/L ferrous iron, but not at 0.025 mg/L ferrous iron, the best estimate threshold would be the geometric mean of 0.025 mg/L and 0.05 mg/L ferrous iron, that is 0.035 mg/L ferrous iron.”

In preparing taste samples for metallic flavor threshold determination, a 100 mg/L iron stock was prepared daily using iron (II) sulfate (Sigma-Aldrich, PA, CAS # 13463-43-g) and deionized water, which was taste- and odor-free. Ferrous solutions were prepared daily by diluting the stock solution with deionized water, which also served as the control. The concentrations tested were 0.005, 0.01, 0.025, 0.05, 0.1, 0.25, 0.5, 1, 2, 4, 5, 10, and 20 mg/L ferrous. Solutions were monitored to prevent the oxidation of ferrous iron to ferric iron. The concentrations were verified using inductively coupled plasma mass spectroscopy (Thermo Electronic Corporation, X-Series ICP-MS, Waltham, MA, USA), following Standard Method 3120B [65].

### 2.7. Data Analyses

Statistical software, JMP 9.0 (SAS, Cary, NC, USA), was used for all data analyses. One-way analysis of variance (ANOVA) and comparison of the means, using Tukey HSD or Wilcoxon/Kruskal Wallis Rank sum test, were performed on the mean salivary lipid oxidation in the test samples. The metal-induced salivary lipid oxidation (SLO) was reported as the arithmetic difference between the measured salivary TBARS (in micromoles/L) in the AS samples without (0 mg/L) and with (180 µM) iron or copper present in the test samples. All statistical analyses were performed at alpha level of 0.05 and results were presented as means ± standard error (SEM). Confidence intervals (95% CI) for the means were reported for all the means. Metallic flavor sensory threshold and beverage consumption data were summarized for comparison of average best estimate metallic flavor sensory threshold and average daily volume intake levels for plain drinking water, sugar-sweetened beverages, other, non-sugar sweetened beverages, and total beverages between younger (18–59 years) and older (60–84 years) participants. To facilitate comparison to existing survey data available from the National Health and Examination Survey (NHANES) [66], the older age group was defined at 60 years of age or older. The F-test for two-sample variance and two sample t-test were used to compare metallic flavor threshold and beverage intake levels between the younger and older age groups. An alpha level of 0.05 was established for all statistical tests. Associations among the continuous variables of drinking water intake level and metallic flavor threshold, sugar-sweetened beverage intake level and metallic flavor threshold, non-sugar-sweetened beverage intake metallic flavor threshold, and total beverage intake level and metallic flavor threshold, were assessed using linear regression analyses.

## 3. Results

### 3.1. Variations in Metal-Induced Lipid Oxidation in Artificial Saliva

**Figure 1:**
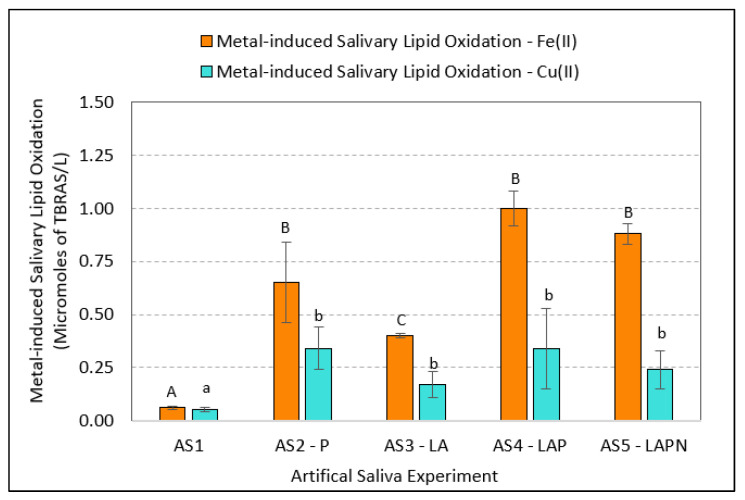
Mean salivary lipid oxidation (SLO), measured in artificial saliva samples using the thiobarbituric acid reactive substances (TBARS) method. Error bars represent 1 standard error from the mean of 4 replicate analyses. Within the same group (i.e., Fe(II) bars in capital letters and Cu(II) bars in small letters), bars with different letters indicate statistical significance (*p* < 0.05) between the compared pairs. Abbreviations: AS = Artificial Saliva; P = Protein; LA = Linoleic Acid; N = Nitrite.

Addition of ferrous iron and/or cupric copper induced some salivary lipid oxidation (SLO) in all artificial saliva solutions, with the exception of the artificial saliva (AS) solution that contained no organic components (AS1); this solution showed very little metal-induced LO (Figure 1). Table 2 provides a summary of the mean metal-induced SLO data.

The highest level of SLO was measured in the artificial saliva samples supplemented with LA and protein (AS4—LAP) and protein (AS2—P), followed by the LA-protein-nitrite-amended samples (AS5—LAPN), and, lastly, the artificial saliva supplemented with LA only (AS3). In samples treated with Fe(II) and Cu(II), there were significant differences between mean SLO levels in the artificial saliva samples (ANOVA for Cu(II): F (4,19) = 5.39; *p* = 0.0007, and for Fe(II): F (4,19) = 16.04; *p* < 0.0001). Follow-up analysis on the means using the Wilcoxon test showed significant differences between AS1 and all other saliva samples (*p* = 0.03) in both Fe(II)- and Cu(II)-treated samples. This was expected, as no or little SLO levels were expected to be measured in the AS1 artificial saliva sample, which contained only inorganic constituents. Additionally, in Fe(II) treated samples, significant differences were measured between mean SLO levels in the AS2—P and AS3—LA, AS3—LA and AS4—LAP, and AS3—LA and AS5—LAPN sample means (*p* = 0.03). In contrast, in the Cu(II)-treated samples, no significant differences were measured between the artificial saliva samples AS2—P, AS3—LA, AS4—LAP, and AS5—LAPN.

### 3.2. Effect of Metal Concentration on Inducing Salivary Lipid Oxidation

**Figure 2:**
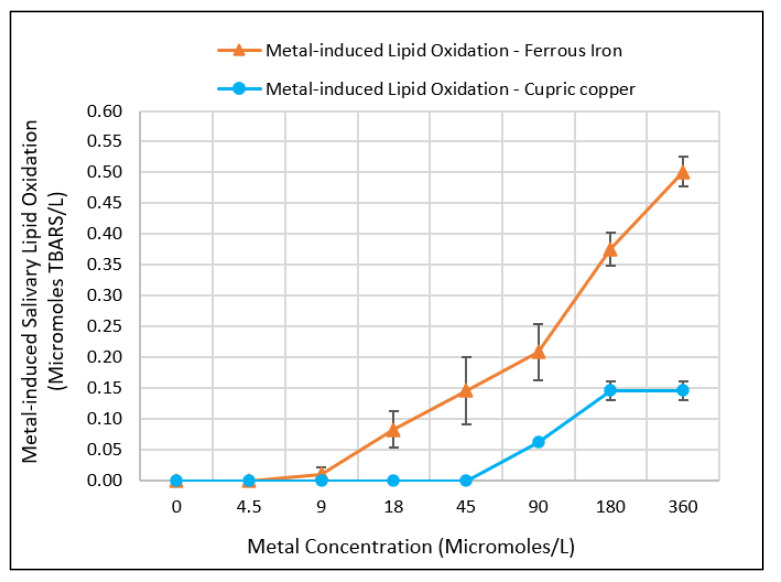
Variations in the mean salivary lipid oxidation (SLO) in artificial saliva versus metal concentration as measured using the thiobarbituric acid reactive substances (TBARS) method. Error bars were constructed using 1 standard error from the mean of 4 replicate analyses.

The addition of Fe(II) and Cu(II) to the artificial saliva samples supplemented with linoleic acid (LA) resulted in an incremental increase of SLO with increasing metal concentration (Figure 2) as measured by the concentration of TBARS in micromoles per liter. At a concentration range of 0 to 360 µM, Fe(II) induced SLO beginning at a concentration of 9 µM (corresponding to 0.5 mg/L Fe) and continued to increase incrementally up to the maximum tested concentration of 360 µM (corresponding to 20 mg/L Fe). Unlike Fe(II), treatment of the LA-supplemented artificial saliva samples with Cu(II) did not induce SLO until the cupric concentration reached 90 µM (corresponding to 5.6 mg/L Cu) and it continued to rise until appearing to level off at about 360 µM (corresponding to 23 mg/L of Cu). In all of the artificial saliva solutions, the pH level was measured at 6.8 ± 1 units and the metals appeared to remain dissolved in solution.

### 3.3. Comparing Beverage Consumptions and Metallic Flavor Sensitivity Levels between Younger and Older Age Groups

**Figure 3:**
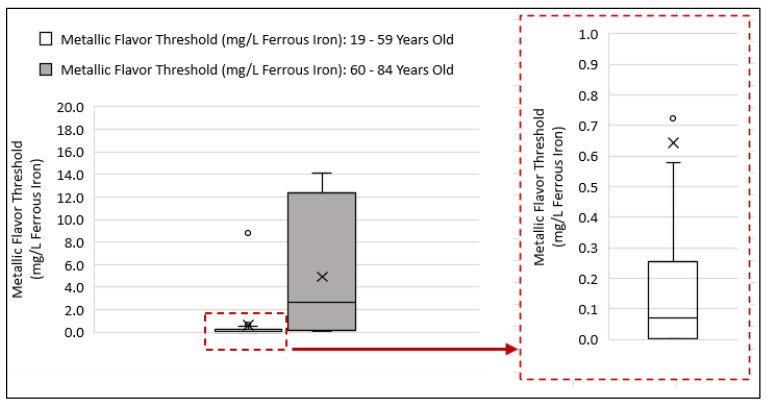
Box plots comparison of metallic flavor thresholds among younger (19–59 years of age) and older participants (60–84 years of age). The dotted red box represents enlarged box plot for the data group category, “Metallic Flavor Threshold (mg/L Ferrous Iron): 19–59 Years Old”. The enlarged box plot to the right displays the same plot, with y-axis scale range of 0 to 1.0 mg/L Ferrous Iron. The open circles displayed on the original box plot (data point 8.77 mg/L Ferrous Iron) and the enlarged portion of the plot (data point 0.724 mg/L Ferrous Iron) represent two outliers in the data set. The lines within each box plot represent the median point in the data group, and the cross (x) represents the mean.

**Figure 4:**
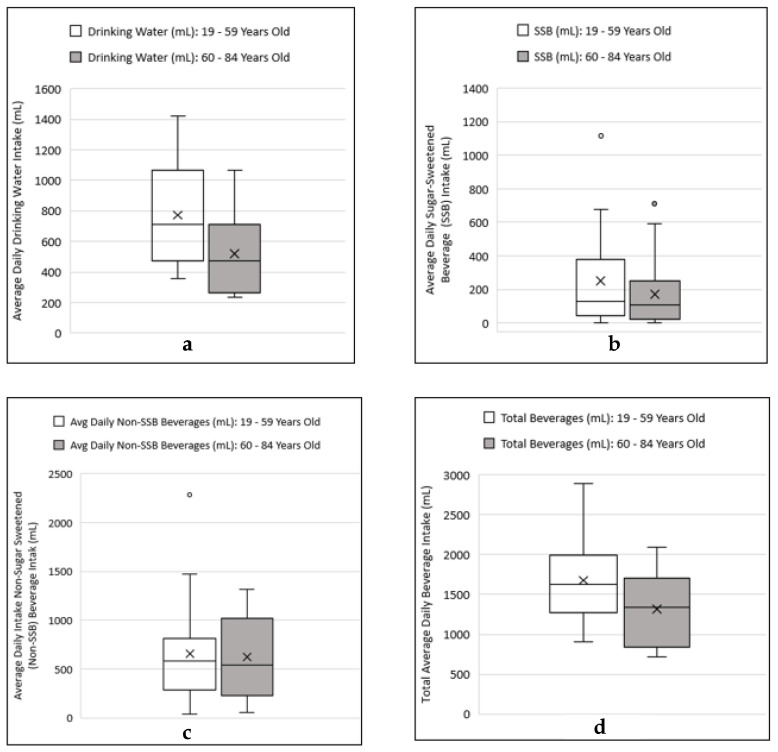
Box plots comparison of average daily intake levels (in milliliters, mL) of consumed beverages. (**a**): drinking water intake; (**b**): sugar-sweetened beverage (SSB); (**c**): non-sugar-sweetened beverage intake; (**d**): total beverages. The circles above the box plots in (**b**,**c**) represent outlier points in the data.

Threshold levels for the metallic flavor of ferrous iron varied greatly among the 33 subjects and ranged from 0.003–14.14 mg/L ferrous. The median threshold level for subjects 19–59 years (*n* = 17) was 0.07 mg/L ferrous iron, and for subjects 60–84 years of age (*n* = 16) the median threshold level was 2.7 mg/L ferrous iron (Figure 3). In comparison, the aesthetic standard, under the US Environmental Protection Agency (USEPA) guideline for metallic flavor of iron, is 0.30 mg/L total iron [1]. The mean metallic flavor threshold levels between the younger (19–59 years of age) and older (60–84 years of age) groups were significantly different (t = −2.76; *p* = 0.012; unequal variance). The median amount for the average daily drinking water intake was higher among the younger group as compared to the older group (Figure 4a), and the difference in the mean drinking water intake levels between the two age groups was significant (t = 2.53; *p* = 0.017; pooled variance). The median amount for the average daily sugar-sweetened beverage intake was higher among the younger group as compared to the older group (Figure 4b), and the difference in the mean sugar-sweetened beverage (SSB) intake levels between the two age groups was not significant (t = 0.86; *p* = 0.40; pooled variance). The median amount for the average daily non-sugar-sweetened (non-SSB) beverage intake was higher among the younger group as compared to the older group (Figure 4c), and the difference in the mean non-SSB intake levels between the two age groups was not significant (t = 0.18; *p* = 0.86; pooled variance). The median amount for the total average daily beverage intake was higher among the younger group as compared to the older group (Figure 4d), and the difference in the mean total average daily beverage intake levels between the two age groups was statistically significant (t = 2.08; *p* = 0.046; pooled variance).

### 3.4. Correlational Relationship between Beverage Consumptions and Metallic Flavor Sensitivity Levels

**Figure 5:**
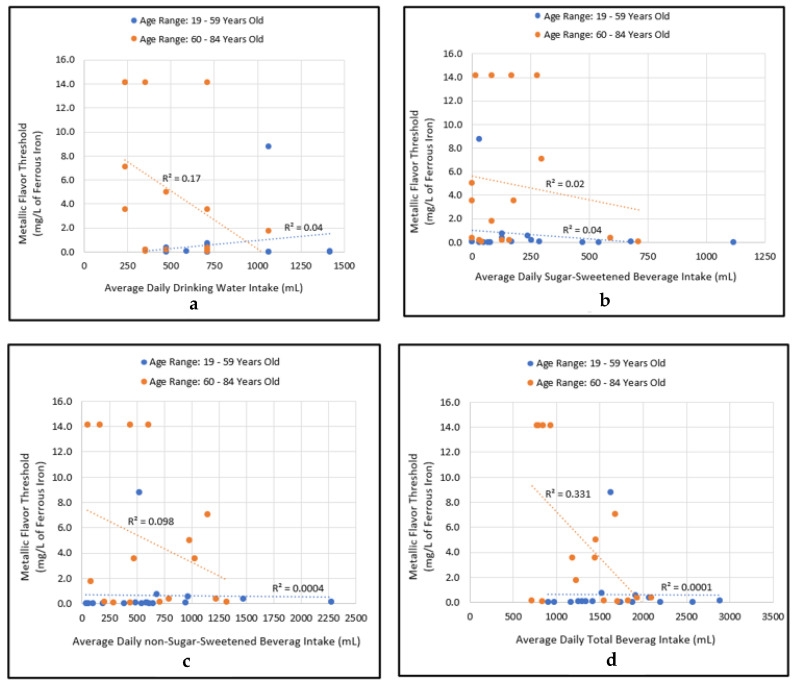
Relationship between metallic flavor threshold and average daily intake levels (in milliliters, mL) of consumed beverages. (**a**): metallic flavor threshold and drinking water intake; (**b**): metallic flavor threshold and sugar-sweetened beverage (SSB) intake; (**c**): metallic flavor threshold and non-sugar-sweetened beverage intake; (**d**): metallic flavor threshold and total beverage intake.

There was no significant correlation between individuals’ average daily drinking water intake levels and sensitivity to metallic flavor of iron (age range 19–59 years: R^2^ = 0.05, *p* = 0.42; age range 60–84 years: R^2^ = 0.17, *p* = 0.11; Figure 5a). Likewise, there were no significant correlations between sugar-sweetened beverages and sensitivity to metallic flavor (age range 19–59 years: R^2^ = 0.04, *p* = 0.43; age range 60–84 years: R^2^ = 0.02, *p* = 0.59; Figure 5b), and non-sugar-sweetened beverage intake levels and sensitivity to metallic flavor (age rang 19–59 years: R^2^ = 0.0004, *p* = 0.94; age range 60–84 years: R^2^ = 0.098, *p* = 0.24; Figure 5c). In contrast, there was a significant correlation between average daily total beverage intake and sensitivity to metallic flavor among individuals in the age group of 60–84 years; however, there was no such correlation among individuals in the age group of 19–59 years (age rang 19–59 years: R^2^ = 0.0001, *p* = 0.97; age range 60–84 years: R^2^ = 0.33, *p* = 0.02; Figure 5d).

Correlational analysis on the relationship between average daily drinking water consumption and sensitivity to metallic flavor of iron on all participants was not significant (age range 19–84 years: R^2^ = 0.06, *p* = 0.16). However, the correlational relationship between average daily total beverage intake and sensitivity to metallic flavor of iron was significant (age range 19–84 years: R^2^ = 0.20, *p* = 0.01).

## 4. Discussion

### 4.1. Assessing the Influence of Salivary Constituents on Metallic Flavor Production Measured by the Thiobarbituric Acid Reactive Substances (TBARS)

The results of the experiments with artificial saliva indicate that the presence of salivary fatty acids and proteins, both individually as well as together, strongly influences the measure of iron- and copper-induced lipid oxidation (LO) in artificial saliva by the TBARS method. Specifically, salivary proteins mucin and alpha-amylase considerably contributed to the measure of lipid oxidation. Although not significant, the presence of nitrite appears to exert an inhibitory effect on salivary LO. When present in saliva, the inhibitory effect of nitrite on LO has been attributed to its conversion to nitric oxide (NO), which can ultimately alter LO pathways through binding with reduced metals such as ferrous iron [67]. Salivary proteins, mucin and alpha amylase, have been recognized for their varying potentials to bind with copper in artificial saliva [21] as well as in vitro using actual human saliva [35]. The binding of copper and iron to salivary protein occurs when these metals are in their free ionic forms, namely ferrous and cupric; this binding in turn can influence their flavor attributes through changing the speciation of metals in saliva [21,35]. Metallic flavor of iron, in its particulate form of stabilized zerovalent nanoparticle, is also believed to be influenced by interaction with salivary fluid as evidenced by lower levels of iron-induced lipid oxidation when compared to that of ferrous iron salt, measured by TBARS in in vitro experiments with human saliva [14]. Another study using artificial saliva showed alpha-amylase to have varying binding capacities for free copper, with the strongest binding occurring at relatively low concentrations of copper (<2.5 mg/L); for example, at pH 6.5 and 2.5 mg/L total copper, the free copper, Cu(II), concentration in artificial saliva was less than 0.1 mg/L compared to about 0.6 mg/L at 5 mg/L total copper [22]. The study also showed that at higher copper concentration (10 mg/L), the binding capacity of alpha-amylase decreased relative to inorganic constituents in saliva. In the same study, similar experiments with the salivary protein mucin demonstrated a considerably higher binding capacity to copper. In relating these findings to the current study, it can be concluded that in the presence of the salivary proteins, Cu(II) becomes less available to induce lipid oxidation, hence, the measure of SLO in Cu(II)-treated artificial saliva (AS) samples was notably lower than that of Fe(II)-treated samples. For the same reasoning, this study supports the prior findings that at the given AS solution pH of 6.8, Fe(II) has a lower binding capacity for the two salivary proteins when compared to copper, hence, resulting in higher availability of iron to induce lipid oxidation in the artificial saliva solutions containing proteins.

Using TBARS as a measure of metal-induced lipid oxidation in saliva, this study shows that the salivary proteins alpha-amylase and mucin, due to their affinity for iron, will compete with salivary lipids to influence production of metallic flavor in the oral cavity, as measured by lipid oxidation using the method of TBARS. While in the case of Cu(II), the presence or absence of the proteins in the artificial saliva did not significantly influence the measure of copper-induced lipid oxidation by TBARS. These results support the previous findings that Cu(II), due to its higher binding capacity to alpha-amylase and mucin than Fe(II), has less of an influence on inducing LO in the protein-amended saliva samples. These findings are informative in terms of health implications, since the composition of proteins and lipids in human saliva has been known to be influenced by diet, among other factors such as age and gender [68,69,70]. Therefore, individuals’ dietary habits may indirectly influence the sensory abilities and inabilities for detecting metallic flavors of iron and copper in drinking water.

### 4.2. Comparing the Fe(II)- and Cu(II)-Induced Salivary Lipid Oxidation by Metal Concentration as Measures of Metallic Flavor Intensity

In relation to metallic flavor attributes for iron and copper, this study shows that by virtue of its higher level of metal-induced LO, upon oral intake, Fe(II) would produce a higher metallic flavor than Cu(II). Reported population thresholds for metallic flavor of ferrous iron range from 0.03 to 0.5 mg/L among individuals with varying sensitivities and ages [6,56], while for cupric copper, reported population thresholds range from 0.4 to 2.5 mg/L [57,58]. Recognizing the findings from previous studies that indicate metallic flavor sensations from foods and beverages are associated with the detection of odorous by-products of LO [4,8,21] upon interaction with salivary fluid, metallic flavor detection thresholds for Fe(II) and Cu(II) are consistent with their respective LO profiles as demonstrated by this study. For Fe(II), LO became measurable by the TBARS method at a concentration of 0.5 mg/L (9 µM), while for Cu(II), LO was measurable at a concentration of 5.7 mg/L (90 µM), approximately 10 times greater than that of Fe(II). In human sensory studies, copper has been shown to produce a more astringent and bitter flavor than iron; meanwhile, iron flavor has been described as more metallic in nature and highly influenced by retronasal odor detection [10]; these attributes are consistent with the observed salivary lipid oxidation profiles of the iron and copper in this study.

In range with previously reported measures of free fatty acids in human saliva [22], the concentration of linoleic acid (LA) in artificial saliva samples for the experiments was 30 mg/L, while the maximum metal concentration tested was about 20 mg/L. As noted earlier, in the concentration range of 0 to 360 µM (equivalent to 20 mg/L Fe(II)), metal-induced LO continued to rise with increasing metal concentration. Conducting similar experiments with artificial saliva at concentrations equal or higher than the amount of fatty acid in the sample would provide additional insights on the LO profiles for the two metals, specifically, identifying the concentration at which LO will cease to rise any further given a fixed reaction time. Additionally, conducting similar experiments with artificial saliva using varying concentrations of salivary lipids and proteins would provide further insights on the role of specific salivary constituents on metal-induced lipid oxidation, as indicators of metallic flavor perception and toxicity profiles. Regardless, it is evident that in the presence of LA, Cu(II)-induced LO is considerably lower than the Fe(II)-induced LO. This difference in pattern of iron- and copper-induced LO has been observed in previous studies [12,35]. As an example, a toxicological study by Repetto and colleagues investigated the role of transition metals, Fe(II), Cu(II), Co(II), and Ni(II), on inducing lipid oxidation in the presence of liposomes, and showed that Fe(II) induced the highest level of LO, followed by Cu(II), while cobalt and nickel had the lowest LO [19]. As used in toxicological studies, the level of lipid oxidation is indicative of the metals’ potentials to produce oxidative stress in biological fluids and tissues, such as in the gut, blood, and oral cavity [71].

### 4.3. Examining Beverage Consumptions and Metallic Flavor Sensitivity Levels between Younger and Older Age Groups

**Figure 6:**
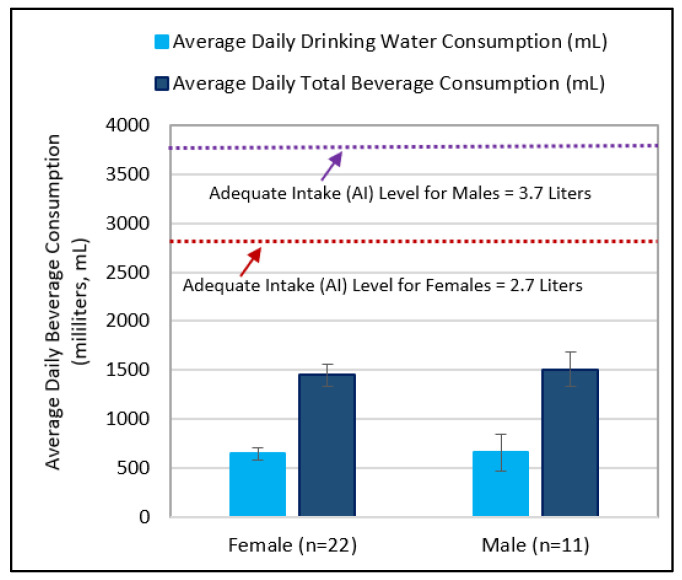
Average daily drinking water and total beverage consumption levels grouped by two genders, males and females, for comparison of the adequate intake (AI) levels. Plotted data represent responses on the validated beverage intake questionnaire (BEVQ) from 33 subjects (22 females), as compared to the AI levels based on the U.S. National Survey Data as established by the Institute of Medicine. Error bars represent 1 standard error from mean. The red and purple arrows in the figure point to the dotted lines that represent the AI levels (2.7 liters and 3.7 liters) of total water intake as recommended by the United States’ Institute of Medicine [43].

This preliminary study examined the beverage consumption patterns and flavor thresholds in adults to identify any relationships between drinking water consumption level, beverage choices, age, and individual taste/flavor sensitivity as assessed by metallic flavor threshold for iron, a commonly occurring flavor complaint among tap water consumers. The most important finding is that among the elderly subjects (60–84 years of age), drinking water and total beverage intake levels are to some degree related to age-associated reduced sensitivity to metallic flavor. Additionally, this study provides additional data in support of previous findings indicating a decline in water intake among elderly population greater than 60 years of age. The water needs of individuals can vary widely based on age, gender, body size, and environmental conditions [40,43]. The adequate intake level (AI) for water from all sources, namely, drinking water, beverages, and foods, is based on estimated median intakes among healthy people in the U.S. national survey data. In terms of drinking water, this study showed that the intake level decline in the elderly group of 60–84 was significant (*p* = 0.017). Likewise, their total water intake from all beverage sources was considerably lower, but not significant (*p* = 0.19) when compared to the younger groups of 19–39 years of age and 40–59 years of age. Additionally, the average daily total water intake levels for both younger and older age groups, ranging from 0.5 to 2.9 L, were near or below the AI levels as established by the Institute of Medicine. When grouped by gender, the total beverage intake levels for males and females were well below the AI levels of 3.7 L and 2.7 L, respectively (Figure 6). The national survey data for adults estimates that water intake from food sources constitute about 20% of the total daily water intake in a typical American diet. Therefore, the actual fluid intake levels are expected to be higher depending on individuals’ diets.

According to the dietary intake panel of the Institute of Medicine, for a healthy person, daily consumption below the AI may not be indicative of additional risk for dehydration and related chronic diseases because a normal hydration status is associated with a wide range of intakes [43]. The composition of body weight is an important factor in variations between individual water needs, since the majority of body water is contained in fat-free body mass [39,40]. Additionally, water needs in individuals may vary based on activity level, diet, and environmental conditions. In older adults, a 3% loss of body weight is considered significant and indicative of a risk for dehydration. Since aging has been associated with loss of body weight, the risk of dehydration is considered to be higher in the elderly than younger adults. Likewise, infants are at a greater risk of dehydration due to their smaller total body weight. However, the risk of dehydration in the elderly is primarily associated with those in assisted living facilities and/or hospitals; otherwise, in healthy older adults, maintenance of body water balance is comparable to that of younger adults [72].

As indicated in this study, drinking water intake declined considerably among elderly adults in the age group of 60–84 years in comparison to younger participants in the age group of 19–59 years (*p* = 0.016; post hoc power = 72%), as did their sensitivity to metallic flavor of iron in drinking water (*p* = 0.008; post hoc power = 78%). More significantly, the decline in total beverage intake among the elderly group correlated with reduced sensitivity to metallic flavor of iron (r = −0.57, *p* = 0.02). Additionally, in this group of elderly adults, total beverage intake was dominated by a higher intake of non-sugar-sweetened beverages (non-SSB), such as 100% fruit juice, unsweetened and/or artificially sweetened coffee or tea, milk, and alcoholic beverages (typically wine), compared to sugar-sweetened beverages (SSB) and plain drinking water. Reduced taste sensitivity may be a factor in consumption of more flavorful beverages instead of plain drinking water. Although previous studies have associated aging with a decline in taste sensitivity [42,43], whether this reduced sensitivity influences the preference for specific beverages remains to be better understood [73,74]. Additional research has shown that impairment of basic taste functions, sweet, sour, bitter, and umami, has implication on dietary intakes of individuals [51,53]. For example, reduced sensitivity to salty taste has been associated with increased intake of salty food and other dietary habits that increase risk of cardiovascular diseases [75]. Other noted factors associated with reduced fluid intake and dehydration risk in older adults are swallowing disorders and decreased olfactory sensation [44]. Oral problems associated with aging, such as the use of dentures and missing teeth, can also discourage or inhibit fluid intake [76].

It is encouraging to note that SSB intake represented the lowest category of total beverage consumption in both age groups as assessed in this study (Figure 4b). However, it should be noted that the participants consisted of mainly university-educated adults, either students and/or current or retired staff and faculty members. Additionally, no data on body mass indices were obtained, although no participant was observationally identified as obese or overweight. Previous researchers have shown that socioeconomic factors have association with higher intake of SSB in low-income households [77]. These findings are consistent with national trends as reported by other researchers [66]. Regardless, it is important to note that consumption of plain water in place of caloric beverages is promoted as a healthy beverage alternative [78], as well as a simple measure to manage weight or even contribute to weight loss [79,80,81].

**Figure 7:**
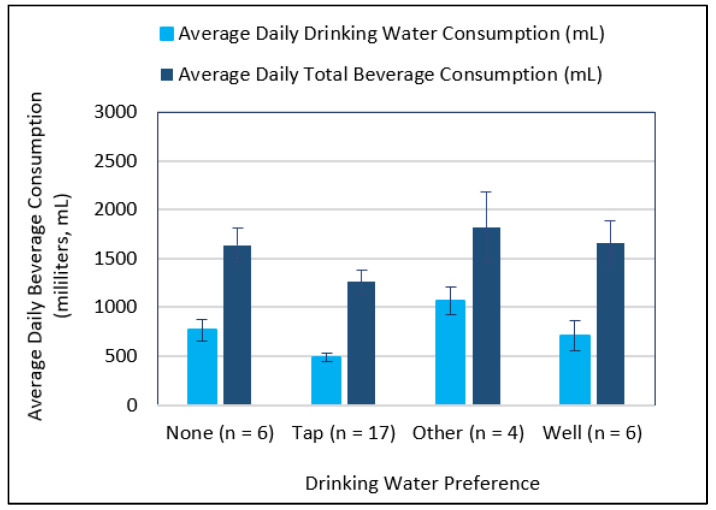
Average daily drinking water and total beverage intake levels according to drinking water preferences. Plotted data represent responses on the validated beverage intake questionnaire (BEVQ) from 33 subjects (22 females). Error bars represent 1 standard error from the means. Drinking water preference category “Other” includes individuals who consumed bottled or filtered water.

Finally, with drinking water being a vital physiological need and widely available resource, understanding the factors that implicate consumer tendencies to drinking water is important. Such information is valuable in order to maintain consumer health and promote water as a beverage for healthy weight management in children as well as adults. Preliminary findings from this research indicate that factors such as water preference and taste/flavor sensitivity may influence the level of consumption. Interestingly, among participants in this study, tap water was indicated as the most preferred water choice, yet was consumed the least (Figure 7).

Previous research has identified multiple factors such as flavor, safety risk perception, prior experience, and trust in water utilities as contributors to consumer’s perception of water quality, which ultimately influences their consumption and choice of water [47,48,82,83,84]. In this study, lower drinking water intake in older adults aged 60–84 years coincided with lower sensitivity to metallic flavor; however, reduced taste sensitivity may not necessarily be associated with reduced consumption levels as indicated by a recent study that reported tap water consumers showed similar taste sensitivity to chlorine but differed in their acceptability of chlorine flavor [85]. Another study identified a gap between consumer preference and consumption habits, indicating that some consumers drink water they do not like simply due to habit [86].

While limited by the scope of sample size, the findings from this study are informative and provide valuable insights for future research. A unique feature of the study is the parallel examinations of metallic flavor threshold, and consumer behaviors in terms of drinking water and total beverage consumption levels. The observed effects of the differences in reduced metallic flavor sensitivity and total beverage consumption levels among younger and older adults were significant in spite of the small sample size, as was the correlational relationship between metallic flavor sensitivity and total beverage consumption levels. These findings indicate that further examination of this relationship in future studies using larger sample sizes is worthy of consideration. Although conducting surveys on drinking water and beverage consumption levels of consumers can be more easily implemented in large cross-sectional surveys, conducting metallic flavor sensory threshold determination studies on large numbers of participants is a time-intensive and difficult undertaking. To overcome such challenges, biochemical markers of metallic flavor sensory perception, such as salivary proteins, lipids, and antioxidant activity, can be utilized to replace or supplement sensory threshold studies.

## 5. Conclusions

As two common off-flavor-producing metals in drinking water, and essential nutrients, iron and copper have potential to cause toxicity at excessive intake levels. Individuals’ varying sensitivity levels and abilities or inabilities to detect metallic off-flavors in water can be influenced by not only age, but also by complex biochemical reactions taking place in salivary fluid. Salivary fluid constituents such as fatty acids and proteins are known to be influenced by diet, age, and health status. Past studies on the relationship between human taste sensitivity and dietary habits have mainly focused on the five basic tastes of sweet, salty, bitter, sour, and umami; however, studies on the relationship between metallic flavor sensitivity and beverage intake patterns are lacking. Findings from this research suggest that the relationship between metallic flavor sensitivity and beverage consumption is more complex and distinctly different from that reported in the literature on relationships between the five basic tastes sensitivities and dietary intake [13,87]. Therefore, further examining this relationship in larger studies, along with use of salivary biomarkers of metallic flavor perception will help to provide a better understanding of the complex nature of human senses of metallic flavor perception and the role that human senses of taste and flavor perception play in influencing beverage consumption. Understanding variables that influence individual beverage consumption patterns is important both in terms of implications on public health as well as consumer acceptability of drinking water as a healthy beverage choice for weight management and wellbeing. While relatively limited in scope, the results of this study indicate that in addition to consumer acceptability and choices, physiological factors such as age-associated variations in individual and/or population taste/flavor sensitivities should be taken into consideration when examining drinking water and overall beverage consumption patterns. This is especially important for reducing dehydration risks, as well as excessive metals exposure risk among susceptible populations, such as the elderly and those impacted by temporary and/or prolonged taste/smell impairments associated with health conditions such as cancer and viral infections associated with the Coronavirus 2019 pandemic (COVID-19) [53,88,89].

This study has practical implications and relevance in multiple disciplines, such as nutrition, environmental engineering, and public health, because drinking water consumption is critical to human health and nutrition, as is access to safe drinking water. Additionally, human senses play an important role in detecting flavor-producing contaminants in drinking water. Therefore, drinking water quality standards must be considerate of varying sensitivity levels of consumers. Biochemical indicators of metallic flavor perception by humans are influenced by saliva composition, which is also influenced by diet. Current research studying the combination of these factors through multidisciplinary perspectives is lacking. This research illuminates the path for future studies exploring the role of metallic flavor sensitivity in environmental exposure assessment studies as well as in consumer nutrition and behavior studies.

## Figures and Tables

**Table 1:** Artificial saliva test sample descriptions and abbreviations.

Abbreviation	Artificial Saliva (AS) Sample Description	Fatty Acid: Linoleic Acid, LA (g/L)	Proteins: Alpha-Amylase + Mucin (g/L)	Metal Concentration (µM) Fe(II) or Cu(II)
AS1	AS	0	0	0, 180
AS2—P	AS + Protein	0	3.78	0, 180
AS3—LA	AS + LA	0.03	0	0, 4.5, 9, 18, 45, 90, 180, 360
AS4—LAP	AS + LA + Protein	0.03	3.78	0, 180
AS5—LAPN	AS + Protein + LA + Nitrite	0.03	3.78	0, 180

**Table 2:** Summary of salivary lipid oxidation (SLO) data.

Artificial Saliva Sample ^(1)^	No. of Replicates (N)	Metal-Induced SLO (Micromoles/L)	SEM ^(2)^	95% Confidence Interval
**Lipid Oxidation Experiments with Fe(II)**				
AS1	4	0.06	0.01	[0.03–0.09]
AS2—P	4	0.65	0.19	[0.04–1.23]
AS3—LA	4	0.40	0.01	[0.36–0.44]
AS4—LAP	4	1.00	0.08	[0.74–1.26]
AS5—LAPN	4	0.88	0.05	[0.71–1.04]
**Lipid Oxidation Experiments with Cu(II)**				
AS1	4	0.05	0.01	[0.04–0.06]
AS2—P	4	0.34	0.10	[0.19–0.50]
AS3—LA	4	0.17	0.06	[0.06–0.27]
AS4—LAP	4	0.34	0.19	[0.04–0.64]
AS5—LAPN	4	0.24	0.09	[0.10–0.38]

(1). See Table 1 for description of abbreviation for artificial saliva (AS) samples. (2). SEM: standard error of the mean.

## Data Availability

Not applicable.
